# Associations between intimate partner violence, childcare practices and infant health: findings from Demographic and Health Surveys in Bolivia, Colombia and Peru

**DOI:** 10.1186/s12889-015-2144-0

**Published:** 2015-08-25

**Authors:** Helga Bjørnøy Urke, Maurice B. Mittelmark

**Affiliations:** Department of Health Promotion and Development, University of Bergen, Christies gate 13, 5020 Bergen, Norway

## Abstract

**Background:**

Child health is significantly poorer in homes with intimate partner violence (IPV). However, a possible link to parental provision of childcare has been neglected.

**Methods:**

Utilizing data from Demographic and Health Surveys, this study examined the association between IPV and illness signs in children 0–59 months in Bolivia (*n* = 3586), Colombia (*n* = 9955) and Peru (*n* = 6260), taking into account socio-demographic factors, childcare and severe child physical punishment. Data were collected in the years 2008, 2010 and 2012 for Bolivia, Colombia and Peru respectively.

**Results:**

The study found weak but persistent effects of IPV on illness signs in Bolivia (OR 1.37, 95 % CI 1.14–1.63) and Peru (OR 1.49, 95 % CI 1.26–1.77), after adjusting for the effects of childcare. These effects were not observed in Colombia.

**Conclusions:**

The results call for a mix of qualitative and quantitative research that can map direct, mediating and moderating patterns of relationships between IPV, childcare practices and child health. Can good childcare mitigate the negative effects of IPV? Can poor childcare exacerbate the negative effects of IPV? Such interactions were not observed in the present study, but should be the focus of much more intensive investigation, to help inform child health promotion. Answers could lead to better interventions to improve child health, and perhaps to tackle IPV.

## Background

Woman-directed intimate partner violence (IPV) committed by one’s domestic partner includes acts of physical aggression, forced intercourse and other forms of sexual coercion, psychological abuse, and controlling behaviours [1]. IPV is ubiquitous across cultures, with men the main perpetrators and women the main victims, but also with obverse manifestations [1, 2]. Because IPV may be exhibited in different ways in different cultures and contexts, there is no globally accepted operational definition. In this paper, IPV is defined as respondents’ self-reports of experiencing acts of emotional, physical and sexual violence by one’s partner.

Increasing recognition and concern over the seriousness and magnitude of woman-directed IPV has triggered research to document its causes, consequences and potential for prevention [1, 3]. IPV is likely to affect all household members including children [4, 5], and IPV’s effects on the child are insidious, having the potential to affect a child’s well-being already in the womb [6].

There is at present a well-developed and compelling literature on IPV’s untoward effects on child mental health [7, 8], and there are suggestions in a much more limited literature that IPV affects physical health. In some studies in Africa and Asia, and very few from Latin America, IPV is significantly associated with low birth weight [9, 10], preterm birth [9], child mortality [11, 12], stunting, wasting and underweight [13–15], diarrhoea [16], illness signs including fever, cough, fast breathing and diarrhoea [17], asthma [18, 19], and acute respiratory tract infection (ARI) [16]. In Silverman et al. [16], children of mothers exposed to IPV were 1.7 times more likely to have had diarrhoea the past two weeks and 1.4 times more likely to have had ARI the past two weeks compared with children of non-exposed mothers. Similarly, in Karamagi et al. [17], children of mothers exposed to IPV were two times more likely to have had diarrhoea in the past two weeks, and 1.8 times more likely to have had signs of illness in the past two weeks compared to children of non-exposed mothers.

It is disconcerting that the findings referred to above were arrived at almost exclusively without taking into account the effects on child health of childcare practices. Some studies have documented a significant association between IPV and the specific childcare practice of vaccination [20]. In studies of IPV’s relationship to the childcare practice of breastfeeding, a lack of high quality research has resulted in insufficient evidence to come to conclusions [20]. In only one of the studies mentioned above was a childcare variable included along with IPV as a potential predictor of child health. In Karamagi, et al.’s study in Uganda [17], exclusive breastfeeding of infants was included as a covariate along with IPV in predicting illness symptoms consisting of fever, cough, fast breathing and diarrhoea; IPV was a significant predictor and breastfeeding was not.

Yount et al. [21] underline important limitations in this literature, including the inconsistent measurement of IPV, the use of small and/or purposive samples, and lack of research on younger children and age-specific studies [21]. There is in addition almost a complete lack of studies from Latin America and the Caribbean, as well as a lack of research that takes into account childcare practices when examining the relationship between IPV and child physical health.

**Fig 1:**
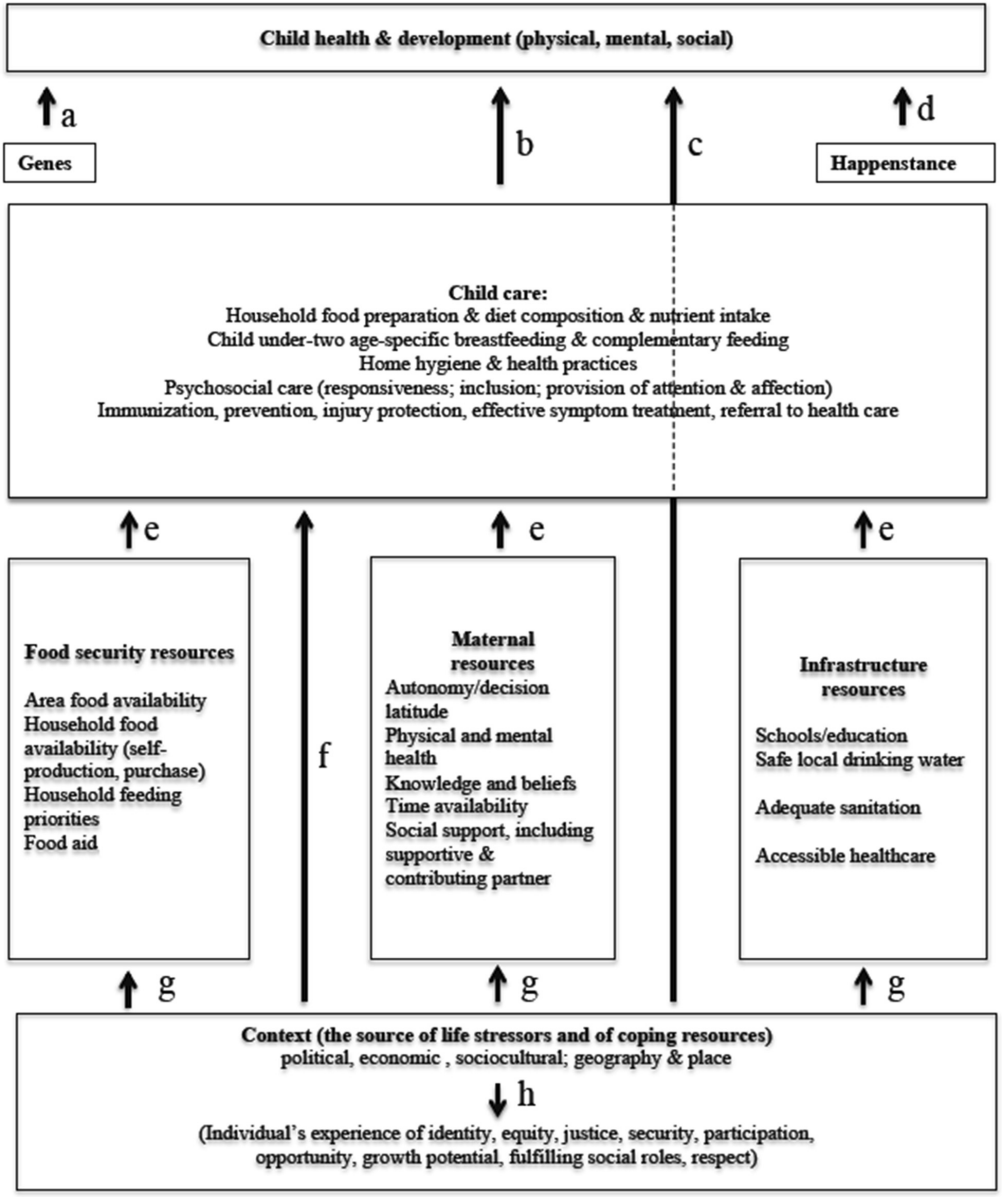
Model of childcare.^a^ Adapted by the Research Unit for Social Determinants of Health in Very Poor Ruralities (MB Mittelmark Director), University of Bergen Research Group *Multicultural Venues in Health, Gender and Social Justice* (http://www.uib.no/rg/mcvenues), from UNICEF, 1990 [44]; Engle Menon and Haddad, 1999 [45]; Smith and Haddad, 2000 [46]

As emphasized by Yount et al. [21], the possible influences and pathways by which IPV might influence a child’s physical health are multiple. Figure 1 presents a framework of childcare [22, 23]. In the framework, child health and development is the ultimate end point. This end point is affected by a range of factors on several levels. The most proximate factors are genes (arrow a), happenstance (arrow d) and childcare (arrow b). Genes and happenstance are not in focus in this study. Childcare is affected by a range of other, more intermediate factors like food security resources, maternal resources and infrastructure resources (arrows e). These all work through childcare to affect child health and development.

`Childcare is prosocial practice but it has some perverse manifestations as domestic violence. The trauma caused by the physical punishment of a child is (usually) intended as childcare. Considering Fig. 1, such physical punishment could confusingly be conceived as a health-related outcome, a childcare factor or a contextual factor, depending on the research perspective.

The most distal factor in the framework is Context. Contextual conditions can work either directly to affect child health and development (arrow c) or indirectly through resources (arrows g) or childcare (arrow f). Arrow h illuminates the social-ecological character of the model, showing that contextual factors influence an individuals’ life in many ways besides health impact (the factors arrow h points to are not in focus in this paper). In this conceptual framework, IPV is a contextual construct in the care environment: a stressor that may affect child health via multiple paths as laid out in the framework. The framework is broadly conceptual and not meant as a guide to the study of IPV and child health in particular. Its utility in the present study is to suggest that research on the IPV/child health link should account for childcare, which is conspicuously lacking in the available literature: childcare may be a critical proximal variable that should be accounted for in any social analysis of child health.

### The Andes region in Latin America

Bolivia, Colombia and Peru share geopolitical history, language, and some common conditions of geographic and social living. They also engage in economic and political cooperation in the form of membership in the Andean Community of Nations trade block (Ecuador is also a member but excluded from the present analysis due to a lack of comparable data). In Peru and Colombia, the percentages of ever-married women who have ever been beaten by a spouse/partner have been estimated at 42.4 % and 44.1 %, respectively [12], and at 47.2 % in Bolivia [24]. Child health in Latin America generally and in the Andes region in particular continues to be a significant public health issue [25–27], underlining the importance of continued research to understand what prevents and promotes child health.

### Study aim

Following from the considerations discussed above, the aim of this study was to examine the association between IPV and child physical health in children 0–59 months in Bolivia, Colombia and Peru, accounting for socio-demographic factors and for childcare practices.

## Methods

The study used nationally representative data in cross-sectional survey designs in the Demographic and Healthy Surveys (DHS) in Bolivia, Colombia, and Peru, collected in 2008, 2010 and 2012, respectively [28–30]. For each country, these were the most recent DHS data available at the time of analysis.

### Sample

A stratified two-stage cluster sampling design was used [31]. The samples used for this study included women 15–49 years and their children 0–59 months for which IPV and illness information was available. This resulted in final samples of 3586, 9955 and 6260 mother-child dyads in Bolivia, Colombia and Peru, respectively.

### Outcome measure

Child health was defined by the mothers’ reports of one or more of the following six illness signs during the two weeks prior to DHS data collection: diarrhoea; blood in stools in case of diarrhoea; fever; cough; short and rapid breaths in case of cough; problems in the chest or running nose in case of cough. Child health was coded as a binary variable with the two categories ‘no signs’ and ‘one or more signs’. In the Bolivia data set, data on fever and problems in the chest or running nose were not available, and hence only the four remaining signs made up the illness symptoms variable for this sample. In using the designation ‘sign’ we follow the medical convention that signs are objective evidence of illness while symptoms are subjective complaints.

### Independent measures

#### Domestic violence

Exposure to IPV was measured through the modified Conflict Tactics Scale (CTS) specially developed for DHS [12]. This is a series of individual questions about acts of emotional, physical and sexual violence such as threatening, slapping, punching, kicking and forcing sex. An example of a question posed is ‘Does your husband/partner ever push you, shake you, or throw something at you?’ with the response categories ‘yes’ and ‘no’, and with a confirmatory response leading to a follow-up question about how many times the last 12 months this happened. IPV was assessed in two ways: 1) ever experienced any violence by current partner, and 2) experienced any violence by current partner the past 12 months. These two variables included emotional violence, less severe and severe physical violence, and sexual violence. The response categories were ‘no’ (0) and ‘yes’ (1). In the Bolivia data set, IPV was only assessed for the past 12 months, hence only this measure was used as the indicator of IPV in these analyses.

Severe physical punishment of child as reported by the mother was included as a domestic violence variable in all multiple regression analyses, operationalised as being beaten on the body or hit with an object (yes/no).

#### Community context

Type of residence (urban/rural) and geographic region were included as community contextual factors (referring to Fig. 1). Categories for geographic region were based on generally accepted and applied categorisations in DHS reports and other literature. For Bolivia, the categories were Valle (valley), Altiplano (high plateau) and Llano (plains). For Colombia, the categories were Bogotá, Pacific, Oriental, Caribbean, Amazonas-Orinoquía and Central. For Peru, the categories were Coast, Andean mountains and Amazon basin.

#### Household and family socioeconomic context

The number of people and number of children under five years residing in the household were treated as continuous variables. Maternal education had the categories *no education, incomplete primary education, complete primary education, incomplete secondary education and complete secondary or higher education.* Household wealth was measured with the standard DHS Wealth Index [32]. Household sanitation was coded improved/unimproved water and toilet facilities as recommended by WHO/UNICEF [33].

#### Childcare

Hygiene practice was measured through a range of questions about hand washing habits, like hand wash after changing diapers and hand wash before feeding the child. Safe disposal of child faeces was determined through criteria developed by WHO/UNICEF [33]. Other indicators of childcare practices were: attitudes towards treatment seeking if the child had illness signs (diarrhoea, fever, vomiting, blood in stools, cough, poor appetite, etc.); whether the child was on track with respect to the country vaccination scheme [28–30]; and whether the child received a minimum acceptable diet based on a 24 h recall as recommended by WHO [34]. All childcare variables were dichotomous with response categories yes/no. Severe physical punishment of child as reported by the mother was included in all multiple regression analyses, operationalised as being beaten on the body or hit with an object (yes/no) by mother or father.

Not all childcare measures were available in all data sets. In Bolivia, data on whether the respondent would take the child to a health facility in case of sickness were not available, and neither were data on hand washing practices. This resulted in some differences in the variables used in the statistical models. Data on minimum acceptable diet for children was collected in sub-samples, resulting in lower sample sizes in some analyses.

### Analysis

Analyses were conducted using SPSS version 19. All analyses were weighted to adjust for the DHS sampling design. Bivariate relationships were tested with the adjusted F statistic to take into account the design effect of the study (for the adjusted F tests, the SPSS complex samples method calculates degrees of freedom as the difference between the number of primary sampling units and the number of strata). Cell-wise standardised residual analysis was used to assess the magnitude of differences between cells’ expected and observed values. The possibility of multicollinearity between independent variables was examined using the Tolerance statistic, and no multicollinearity was observed at the level Tolerance ≤ 0.1 [35]. Multiple binary logistic regression was used to test the relationship of predictor variables to the outcome variable. The construction of the regression models was based on two considerations – to include key factors as proposed in the conceptual framework in Fig. 1, and to include variables that were statistically significantly associated with the outcome measure in bivariate analyses. The approach to constructing the models was the same for all three samples. Variables were included in blocks starting with maternal age and IPV, followed by socio-demographic and childcare variables. Moderating effects involving IPV and the contextual and childcare variables were tested with interaction terms in regression models. The study of possible mediating effects of childcare in the relationship between IPV and illness signs was planned but not conducted for reasons clarified in Results.

### Ethics

Informed consent and anonymity were assured before and during data collection according to the applicable ethics protocols [28–30]. The questionnaires and protocols were reviewed and approved by the Macro Institutional Review Board and relevant in-country authorities. The implementation of the domestic violence module in the DHS follows WHO recommendations for ethical collection of data on domestic violence [12].

## Results

Descriptive characteristics of the outcome, main predictor variables and control variable are provided in Table 1. In Bolivia, 50 % of children 0–59 months were reported to have had one or more illness signs the past two weeks. The proportions with illness signs for Colombia and Peru were 55 % and 46 %, respectively.

**Table 1:** Descriptive characteristics of dependent, main predictor and control variables

		Bolivia 2008 (*N* = 3586)			Colombia 2010 (*N* = 9955)			Peru 2012 (*N* = 6260)		
		N (%)^a^	Mean	SD	N (%)^a^	Mean	SD	N (%)^a^	Mean	SD
Dependent variable										
Illness signs	No	1796 (50.1)			5051 (45.2)			3407 (54.4)		
	Yes	1790 (49.9)			6128 (54.8)			2853 (45.6)		
Main predictor variables										
Experienced any IPV past 12 months by current partner	No	2201 (61.4)			6040 (60.7)			4824 (77.1)		
	Yes	1385 (38.6)			3915 (39.3)			1436 (22.9)		
Ever experienced any IPV by current partner	No	NA			5418 (54.4)			3670 (58.6)		
	Yes				4537 (45.6)			2590 (41.4)		
Severe physical punishment of child	No	2997 (83.6)			7680 (77.1)			4264 (68.1)		
	Yes	586 (16.3)			2275 (22.9)			1996 (31.9)		
	Missing	3								
Control variable										
Maternal age	15–49	3586	25.19	5.479	9955	27.95	6.825	6260	29.99	6.946

N.A. indicates information not available

^a^Valid percent, missing ignored

In Bolivia, only experience of IPV the past 12 months by current partner was available, and 39 % reported experiencing any IPV the past 12 months. In Colombia, 46 % of the women reported ever experiencing any IPV by their current partner, and 39 % reported experiencing any IPV by their current partner the past 12 months. In Peru, 41 % of the women reported ever experiencing any IPV by their current partner, and 23 % reported experiencing any IPV by their current partner the past 12 months. The prevalence of severe physical punishment of child was 16 %, 23 % and 32 % in Bolivia, Colombia and Peru, respectively. Other descriptive results are presented in Table 2 (context and resource variables) and Table 3 (care variables).

**Table 2:** Descriptive characteristics of contextual and resource variables for samples Bolivia DHS 2008, Colombia DHS 2010 and Peru DHS 2012

		Bolivia 2008 (*N* = 3586)			Colombia 2010 (*N* = 9955)			Peru (*N* = 6260)		
		N (%)^a^	Mean	SD	N (%)^a^	Mean	SD	N (%)^a^	Mean	SD
Contextual variables										
Residence	Urban	2080 (58.0)			6359 (63.9)			3600 (57.5)		
	Rural	1506 (42.0)			3596 (36.1)			2660 (42.5)		
Geographic region^b^	Altiplano/Bogotá/Andes	1308 (36.5)			451 (4.5)			2518 (40.2)		
	Valle/Pacífica/Coast	1109 (30.9)			1285 (12.9)			2028 (32.4)		
	Llano/Oriental/Amazonas	1169 (32.6)			1563 (15.7)			1714 (27.4)		
	Caribbean				2136 (21.5)					
	Amazonas-Orinoquía				2135 (21.4)					
	Central				2385 (24.0)					
Number of household residents		3586	5.04	2.118	9955	5.56	2.385	6260	4.99	1.774
Number of children 0–59 months residing in household		3586	1.74	.783	9955	1.62	.802	6260	1.33	.597
Resource variables										
Household wealth	Fifth (Richest)	717 (20.0)			1942 (19.5)			880 (18.0)		
	Fourth	717 (20.0)			2013 (20.2)			933 (19.1)		
	Third	717 (20.0)			2006 (20.2)			1031 (21.1)		
	Second	717 (20.0)			1999 (20.1)			1021 (20.9)		
	First	718 (20.0)			1995 (20.0)			1023 (20.9)		
Maternal education	Secondary/higher	1324 (36.9)			3954 (39.7)			2835 (45.3)		
	Incomplete secondary	560 (15.6)			2586 (26.0)			1194 (19.1)		
	Complete primary	260 (7.2)			1508 (15.1)			913 (14.6)		
	Incomplete primary	1366 (38.1)			1593 (16.0)			1111 (17.7)		
	No education	76 (2.1)			314 (3.2)			207 (3.3)		
Sanitation	Improved	988 (59.5)			7165 (72.0)			3201 (52.2)		
	Unimproved	672 (40.5)			2790 (28.0)			2933 (47.8)		
	Missing	1926						126		

N.A. indicates information not available

^a^Valid percent, missing ignored

^b^Country specific regions are presented and distinguished by /

N.A. indicates information not available

**Table 3:** Descriptive characteristics of childcare variables for samples Bolivia DHS 2008, Colombia DHS 2010 and Peru DHS 2012

		Bolivia 2008 (*N* = 3586)			Colombia 2010 (*N* = 9955)			Peru (*N* = 6260)		
		N (%)^a^	Mean	SD	N (%)^a^	Mean	SD	N (%)^a^	Mean	SD
Childcare variables										
Hand wash after using the toilet	Yes	N.A.			N.A.			4186 (66.9)		
	No							2073 (33.1)		
	Missing							1		
Hand wash after changing diapers/cleaning baby	Yes	N.A.			10,559 (94.5)			1292 (20.6)		
	No				617 (5.5)			4967 (79.4)		
	Missing							1		
Hand wash before preparing meals	Yes	N.A.			N.A.			4487 (71.7)		
	No							1772 (28.3)		
	Missing							1		
Hand wash before serving food	Yes	N.A.			N.A.			1349 (21.6)		
	No							4910 (78.4)		
	Missing							1		
Hand wash before eating	Yes	N.A.			N.A.			4578 (73.1)		
	No							1681 (26.9)		
	Missing							1		
Hand wash before feeding child	Yes	N.A.			N.A.			1863 (29.8)		
	No							4396 (70.2)		
	Missing							1		
Disposal of child stools	Safe	1293 (36.2)			5182 (48.7)			3287 (53.2)		
	Unsafe	2274 (63.8)			5454 (51.3)			2888 (46.8)		
	Missing							1		
Take child to health facility if it doesn’t eat or drink	Yes	N.A.			N.A.			68 (1.1)		
	No							6191 (98.9)		
	Missing							1		
Take child to health facility if becomes sicker	Yes	N.A.			N.A.			416 (6.6)		
	No							5843 (93.4)		
	Missing							1		
Take child to health facility if diarrhoea/fever/vomits	Yes	N.A.			10,507 (94.0)			5837 (93.3)		
	No				669 (6.0)			422 (6.7)		
	Missing				3					
Take child to health facility if it has short, rapid breaths	Yes	N.A.			1508 (13.5)			1096 (17.5)		
	No				9668 (86.5)			5163 (82.5)		
	Missing				3			1		
Take child to health facility if it has cough/difficulty breathing	Yes	N.A.			3402 (30.4)			2677 (42.8)		
	No				7774 (69.6)			3582 (57.2)		
	Missing				3			1		
Take child to health facility if it has blood in stools	Yes	N.A.			10,934 (97.8)			196 (2.5)		
	No				242 (2.2)			7615 (97.5)		
	Missing				3					
Minimum acceptable diet 6–59 months^b^	Yes	898 (59.2)			2619 (44.0)			2699 (76.8)		
	No	620 (40.8)			3340 (56.0)			833 (23.6)		
	Missing	2068			3996			2728		
Complete immunization 0–59 months	Yes	2812 (78.6)			6480 (71.8)			3754 (67.4)		
	No	765 (21.4)			2547 (28.2)			1813 (32.6)		
	Missing	9			928			693		

N.A. indicates information not available

^a^Valid percent, missing ignored

^b^Minimum acceptable diet was only available for a subset of the sample and excluded children 0–6 months

### Bivariate analyses

Results of bivariate analyses are presented in Table 4, and key findings are highlighted in the text below.

**Table 4:** Bivariate association of possible predictors with reported signs of illness in children 0–59 months. DHS Bolivia 2008, Colombia 2010, and Peru 2012

	Bolivia 2008 (*N* = 3586)		Colombia 2010 (*N* = 9955)		Peru 2012 (*N* = 6260)	
	Illness signs		Illness signs		Illness signs	
	Adjusted F	df	Adjusted F	df	Adjusted F	df
Domestic violence						
Ever any IPV	-	-	4.105*	1, 3334	30.478***	1, 1341
IPV past 12 months	13.105***	1, 928	2.891	1, 3483	20.993***	1, 1341
Severe physical punishment of child	2.266	1, 928	3.339	1, 3334	9.748**	1, 1341
Contextual factors						
Urban/rural residence	9.745**	1, 928	2.975	1, 3334	0.580	1, 1341
Geographic region	4.946**	1, 928	18.636***	4, 13393	22.975***	2, 2568
Resources						
Household wealth	2.787*	4, 3657	4.504***	4, 12834	0.668	4, 5073
Maternal education	1.553	4, 3678	0.775	4, 13194	2.235	4, 5240
Sanitation	0.512	1, 928	2.552	1, 3334	1.673	1, 1341
Care						
Hand wash after using the toilet	-	-	-	-	0.010	1, 1341
Hand wash after changing diapers	-	-	0.606	1, 3334	8.239**	1, 1341
Hand wash before preparing meals	-	-	-	-	0.593	1, 1341
Hand wash before serving food	-	-	-	-	0.684	1, 1341
Hand wash before eating	-	-	-	-	1.214	1, 1341
Hand wash before feeding child	-	-	-	-	2.599	1, 1341
Disposal of child stools	0.065	1, 928	1.405	1, 3334	9.532**	1, 1341
Take child to health facility if it doesn’t eat or drink	-	-	1.276	1, 3334	0.831	1, 1341
Take child to health facility if becomes sicker	-	-	1.277	1, 3334	0.169	1, 1341
Take child to health facility if diarrhoea/fever/vomits	-	-	5.060*	1, 3334	1.058	1, 1341
Take child to health facility if it has short, rapid breaths	-	-	0.587	1, 3334	10.797***	1, 1341
Take child to health facility if it has cough/difficulty breathing	-	-	7.845***	1, 3334	3.806	1, 1341
Take child to health facility if it has blood in stools	-	-	1.870	1, 3334	0.001	1, 1341
Minimum acceptable diet	3.714	1, 928	0.275	1, 3334	0.247	1, 1341
Complete immunization age specific	0.070	1, 928	0.853	1, 3334	0.009	1, 1341

*Association is statistically significant at *p* < .05

**Association is statistically significant at *p* < .01

***Association is statistically significant at *p* < .005

### Illness signs, IPV, and severe child physical punishment

In Bolivia and Peru, experiencing IPV the past 12 months was associated with illness signs. In both samples, the prevalence of illness was greater than expected by chance among children of mothers reporting experiencing IPV the past 12 months (Standard residual (std. resid.) = 3.5 and 4.0 in Bolivia and Peru, respectively), and lower than expected by chance among children of mothers reporting no IPV the past 12 months (std. resid. = −2.7 and −2.2 in Bolivia and Peru respectively). In Colombia and Peru, ever having experienced IPV by the current partner was associated with illness signs. In both countries, illness sign prevalence was greater than expected among children of mothers reporting IPV (std. resid. = 1.9 and 3.8 in Colombia and Peru, respectively), and lower among children of mothers reporting no IPV (std. resid. = −2.1 and −3.2 in Colombia and Peru, respectively).

Severe physical punishment of the child was associated with illness signs in the Peru sample only. The prevalence of illness signs was higher than expected by chance among children experiencing severe physical punishment (std. resid. = 2.1), and lower than expected by chance among children not experiencing severe physical punishment (std. resid. = −1.5).

### Illness signs and childcare variables

None of the childcare variables available in the Bolivia sample was associated with illness signs. In the Colombia sample, taking children to a health facility in case of diarrhoea, fever or vomiting and in case of cough or difficulty breathing was associated with illness signs. The prevalence of illness signs was greater than expected among children of mothers reporting no treatment seeking in case of diarrhoea, fever or vomiting (std. resid. = 2.9), and lower among children of mothers reporting treatment seeking (std. resid. = −0.7). Conversely, the prevalence of illness signs was greater than expected among children of mothers who reported treatment seeking in case of cough or difficulty breathing (std. resid. = 2.8), and lower among children of mothers reporting no treatment seeking in case of cough or difficulty breathing (std. resid. = −1.8). In Peru, hand wash after changing diapers was associated with illness signs. The prevalence of illness signs was greater than expected among children of mothers reporting washing hands after changing diapers (std. resid. = 1.8), and lower among children of mothers not reporting this hygiene practice (std. resid. = −0.9). Disposal of child stools was associated with illness signs. The prevalence of illness signs was greater than expected among children of mothers reporting unsafe disposal of child stools (std. resid. = 1.9) and lower among children of mothers reporting safe disposal of child stools (std. resid. = −1.8). Lastly, taking child to a health facility in case of short, rapid breathing was associated with illness signs. The prevalence of illness signs was greater than expected among children of mothers reporting that they would take the child to a health facility in case of short, rapid breaths (std. resid. = 4.1), and lower among children of mothers reporting not taking the child to a health facility in case of short, rapid breaths (std. resid. = −1.9).

### Regression analyses

Results of logistic regression analyses are presented in Table 5. Not shown are preliminary models in which the variables were entered in sequential blocks. The models in Table 5 are the final and fully adjusted models in which context and care variables as laid out in the conceptual framework were included.

**Table 5:** Illness signs in children regressed on Intimate Partner Violence (IPV) and contextual, resource and care factors

	Bolivia^a^				Colombia^a^				Peru^a^		
	B	O.R.	C.I.		B	O.R.	C.I.		B	O.R.	C.I.
Independent measures:											
Maternal age (continuous)	−0.03	**0.97**	**0.96**, **0.99**		−0.02	**0.98**	**0.97**, **0.99**		−0.01	0.99	0.98, 1.00
Ever any IPV (ref = No)^b^	0.31	**1.37**	**1.14**, **1.63**		−0.09	1.10	0.98, 1.23		0.40	**1.49**	**1.26**, **1.77**
Severe child physical punishment (ref = No)	−0.14	1.15	0.91, 1.47		−0.17	**1.18**	**1.03**, **1.35**		-.18	1.19	0.99, 1.43
Urban/rural residence (ref = Urban)	0.41	**1.50**	**1.09**, **2.07**		−0.39	**0.68**	**0.55**, **0.83**		−0.21	0.81	0.64, 1.03
Region (ref = Altiplano/Bogotá/Sierra)											
Valle/Oriental/Coast	0.07	1.08	0.87, 1.33		0.05	1.05	0.81, 1.35		0.19	1.21	0.98, 1.48
Llano/Pacífica/Amazon basin	0.42	**1.52**	**1.19**, **1.95**		0.30	1.27	0.98, 1.64		0.49	**1.63**	**1.29**, **2.05**
Amazonas-Orinoquía					0.24	**1.34**	**1.04**, **1.74**				
Central					0.40	**1.49**	**1.17**, **1.88**				
Caribbean					0.64	**1.90**	**1.48**, **2.43**				
Maternal education (ref = Complete secondary/higher)											
Incomplete secondary	0.04	1.04	0.77, 1.41		0.06	0.94	0.81, 1.10		−0.09	0.92	0.72, 1.16
Complete primary	−0.09	0.91	0.62, 1.33		−0.12	0.89	0.73, 1.08		−0.29	0.75	0.57, 0.99
Incomplete primary	0.00	1.00	0.78, 1.29		−0.14	0.87	0.71, 1.07		−0.25	0.78	0.59, 1.04
No education	−0.21	0.81	0.41, 1.63		−0.14	0.87	0.59, 1.27		0.33	1.39	0.71, 2.73
Household Wealth Index (ref = Fifth/Richest)											
Fourth	−0.11	0.89	0.69, 1.16		0.14	1.15	0.97, 1.37		0.21	1.22	.92, 1.61
Third	−0.10	0.90	0.67, 1.22		0.27	**1.30**	**1.07**, **1.59**		0.30	**1.34**	**1.02**, **1.76**
Second	0.03	0.97	0.62, 1.50		0.46	**1.58**	**1.23**, **2.03**		0.26	1.28	0.93, 1.75
First	−0.04	0.96	0.61, 1.51		0.33	**1.38**	**1.04**, **1.85**		0.46	**1.57**	**1.12**, **2.21**
Hand wash after cleaning baby/changing diapers (ref = Yes)					0.05	1.05	0.81, 1.36		−0.13	0.87	0.70, 1.09
Handling child’s stools (ref = Safe)	−0.05	0.96	0.80, 1.14		−0.17	**0.85**	**0.75**, **0.96**		0.10	1.11	0.93, 1.33
Take child to health facility if ill with diarrhoea/fever/vomit (ref = Yes)		N.A			0.20	1.23	0.91, 1.65		0.17	1.19	0.83, 1.70
Take child to health facility if child has cough (ref = Yes)		N.A			−0.11	0.90	0.79, 1.02		−0.11	0.90	0.76, 1.08
Take child to health facility if child has short, rapid breaths (ref = Yes)		N.A			−0.12	0.89	0.74, 1.06		−0.18	0.83	0.68, 1.02
Complete immunization (ref = Yes)	0.05	1.05	0.85, 1.30		−0.04	0.96	0.84, 1.09		−0.10	0.91	0.75, 1.09

*OR* indicates odds ratio, *CI* indicates confidence interval, *ref* indicates reference category

Odds Ratios with Confidence Intervals not including 1 are indicated in bold

^a^Due to data availability limitations, for Bolivia, IPV past 12 months is predictor and not ever experienced any IPV by current partner ﻿r2 model fit estimates = 0.03–0.04, 0.04–0.05, and 0.03–0.05 for Bolivia, Colombia and Peru respectively

^b^Due to data availability limitations, for Bolivia, IPV past 12 months is predictor and not ever experienced any IPV by current partner

In the adjusted model in Bolivia, having experienced any IPV by current partner the past 12 months was statistically significantly associated with illness signs. Further, geographic region, residence and maternal age were statistically significantly associated with illness signs. Children living in the Llano region had increased odds of illness signs of 1.52 compared with those living in the Altiplano region. No interaction effects were observed. The fit of the final model was poor as judged by r^2^ = 0.03–0.04.

Regression analyses using the Colombia sample (Table 5) found no statistically significant effect of ever experiencing IPV by current partner in either unadjusted or adjusted models. Of the community context variables, residence and geographic region were statistically significantly associated with illness signs in the adjusted model. Rural residence had a protective effect on illness signs and living in all geographic regions except the Oriental and Pacific regions, increased the odds of child morbidity compared to living in Bogotá (see Table 5 for details). Of the household contextual variables, the three lowest wealth quintiles of the Wealth Index were statistically significantly associated with increased likelihood of illness signs. Of the care variables, only safe handling of child’s stools was statistically significantly associated with illness signs. No interaction effects were observed. The fit of the final model was poor as judged by r^2^ = 0.04–0.05.

For the Peru sample (Table 5), a statistically significant effect on illness signs was observed for ever experiencing IPV by current partner after adjusting for contextual and care variables. In addition, geographic region and wealth were statistically significantly associated with illness signs. Children living in the Amazon basin were 1.6 times more likely to have illness signs compared to children living in the Andean region. No interaction effects were observed. The fit of the final model was poor as judged by r^2^ = 0.03–0.05.

The same analyses were repeated in the Colombia and Peru data with the variable ‘experienced any IPV the past 12 months by current partner’ as the main predictor. The results were almost the same as the results presented in Table 5 and will therefore not be described or discussed further.

Child diet was not available for the entire sample of mother-child dyads with IPV and illness data, and was therefore left out of the reported regression analysis. However, considering the importance of child diet as childcare factor, a separate regression analysis was performed including child minimum acceptable diet for children 6–59 months. The relationship between IPV and child illness signs remained statistically significant in Bolivia and Peru, and insignificant in Colombia with the inclusion of the diet variable. Minimum acceptable diet was not statistically significantly associated with illness signs (Bolivia: OR 1.27, 95 % CI 0.97–1.67; Colombia: OR 1.17, 95 % CI 1.00–1.36; Peru: OR 0.85, 95 % CI 0.63–1.14).

The relationships between IPV and illness signs were weak. Therefore, mediation analyses were not undertaken, as had been planned originally, since any mediation effects are negligible in the present data.

## Discussion

In this study, close to 50 % of children in Bolivia, Colombia and Peru were reported by their mothers to have had signs of illness during the two weeks prior to the survey. Further, from 23 % (Peru) to 39 % (Colombia and Bolivia) of women living with a partner reported experiencing IPV the past 12 months. The prevalence of ever experiencing IPV was higher in Colombia and in Peru compared to other estimates of the America region as a whole [36].

The study found significant associations of IPV to illness signs in Bolivia and Peru, after accounting for childcare factors and the infliction of physical punishment. Indeed, severe physical punishment of children was a significant risk factor for child illness signs in the fully adjusted models. In these samples, violence directed at the child, compared to exposure to IPV, bore the strongest relationship to child health. There is need for more research on the child health effects of indirect *and* direct exposure to violence [5]. As already noted, the punishment of a child is a childcare behaviour in the eyes of most caregivers. In this study, we have not clustered it with the childcare variables, because of its kinship with IPV as a stressor.

The lack of association between IPV and illness signs in Colombia (independent of severe physical punishment of the child) deserves consideration. Colombia had somewhat higher public expenditure on health in 2010 (5.5 %) compared to Bolivia (3.7 %) and Peru (2.8) as reported by the UNDP Human Development report from 2013 [37]. This might result in a buffer effect wherein IPV triggers the provision of better health care in Colombia. However, this is merely speculation, and it is cautionary to note that on some public health measures like immunisation coverage, the three countries hardly differ [37].

### Contextual variables

In this study, children residing in rural areas in Bolivia were more likely to be reported having had illness signs the past two weeks. Conversely, children residing in rural areas in Colombia were less likely to be reported having had illness signs the past two weeks. The contradictory findings may be due to country specific factors like poorer living conditions for rural residents in Bolivia and improved living conditions for rural residents in Colombia. This possibility could be addressed empirically with DHS data by comparing the socioeconomic status of urban and rural families in both countries. It is also possible that the urbanisation experience in the two countries differs with regard to health effects; generally, urban compared to rural residence jeopardizes health, particularly in the poorer segments of a population [38]. The LAC region has experienced high rates of urbanisation the past decades and the challenges of developing sufficient urban infrastructure have been significant [38]. Better success in Bolivian urbanisation compared to Colombian urbanisation, and/or better rural services in Colombia than in Bolivia, are possible explanations for the present findings, but this cannot be explored in the DHS data.

In all three samples, compared to residing in the geographic region with the lowest prevalence of illness signs, living in most other geographic regions was a risk factor for child illness signs. This suggests that a host of contextual factors help determine child health, which is certainly not a novel suggestion. However, there is a tendency for public health work to use national campaign models, and these results suggest that local, context-fitted child health interventions might be more prudent.

A risk effect on child health of being in the three poorest wealth quintiles, compared to being in the richest wealth quintile, was observed in Colombia, and similarly in the middle and poorest wealth quintiles in Peru. This is consistent with others’ observation of a relationship between socioeconomic status and health [26, 39]; yet in this study, maternal education was not related to child health, as might be expected. The data in Table 2 suggests that the examination of maternal education in relation to child health should be approached with caution. The level of low education in Bolivia is twice that of Colombia and Peru, while the level of higher education in Peru is higher than in the other two countries. Socioeconomic and/or cultural differences in education opportunities for women in these countries might account for the present findings.

This study analysed a large range of the relevant variables in the DHS data. Despite this, the variance in child health explained by the final models was very modest, indicating that the variables included are poor predictors of child illness signs. This could be due to the way these variables are measured, or that other variables not available in the DHS data are more important in explaining illness signs. With very few exceptions, DHS do not collect data on mental- and psychosocial health, social integration, social support, or interpersonal relationships in families, which are shown both to be related to living conditions [40] and to have effects on physical health [14, 41, 42]. In addition, the DHS does not measure respondents’ exposure to health education campaigns and other efforts by government and non-governmental organisations to improve child health. We suggest that large surveys like the DHS could make significant contributions to understanding child health, if such data were included in future survey rounds.

### Limitations and recommendations

Although IPV was measured with a validated, standard scale, this scale has weaknesses. Critics argue that it does not distinguish between initiated violence and self-defence, it weights all actions of violence equally, and it ignores the meaning attached to the violent action, all of which might influence an IPV experience [43]. Acknowledging that this measure might represent an oversimplification of IPV, the aim of this study was to test whether a report of any type of IPV is related to illness signs in representative populations, and not to study the meanings attached to the violence.

The number of variables included in this analysis was limited, and inclusion of other contextual or care variables could have altered the observed association between IPV and illness signs. Future studies should aim at giving closer attention to a larger range of maternal and childcare factors. Further, comparability of the country specific analyses is not straight forward, as these were not identical. Due to missing variables in some of the datasets (e.g., lack of many care variables in the Bolivia data), it is difficult to assess the role of care in the relationship between IPV and illness signs in the Bolivia sample. Lastly, no analysis was conducted examining mediating mechanisms in the relationship of IPV to illness signs. To better understand and test the framework in Fig. 1, future studies should examine the indirect effects of IPV through resources and care factors on various measures of child physical health, using appropriate mediation analyses. This assumes that reasonably large main effects are first detected, since mediation effects are partitions of main effects.

## Conclusion

This study contributes to a limited literature on IPV, childcare and health. The results of the study indicate a significant relationship between IPV and illness signs, and between severe child physical punishment and illness signs. As suggested by the framework that guides this study, additional research is needed to explore the pathways through which the relationships between IPV, childcare and child health are connected. IPV may have a direct effect on child health due to the trauma of being exposed to acts of IPV, even if the child is not a direct victim of violence. IPV may also foment a culture of violence in the home, to which the child may also fall victim. On the other hand, there is some evidence that IPV may have a reduced negative effect on child health, in situations where a victimised primary caregiver is especially protective of those she cares for. This study provides evidence that IPV has a direct effect on child health independent of childcare. Can good childcare mitigate the negative effects of IPV? Can poor childcare exacerbate the negative effects of IPV? Such interactions were not observed in the present study, but should be the focus of much more intensive investigation, to help inform child health promotion. A more comprehensive mapping of childcare environments and childcare practices is called for to develop knowledge about protective and risk factors of child health and development. Results could lead to better interventions to improve child health, and perhaps to tackle IPV.

## Abbreviations

IPV: Intimate partner violence
DHS: Demographic and Health Surveys
CTS: Conflict Tactics Scale
